# Management of Cisplatin-Induced Encephalopathy: A Case Report and Literature Review

**DOI:** 10.7759/cureus.62176

**Published:** 2024-06-11

**Authors:** Fadoua Jebrouni, Hanan Bailal, Mouhsine Omari, Kaouthar Khater, Asmae Bali, Ouissam Al Jarroudi, Sami Aziz Brahmi, Said Afqir

**Affiliations:** 1 Medical Oncology, Mohammed I University, Oujda, MAR; 2 Medical Oncology, Mohammed VI University Hospital, Faculty of Medicine and Pharmacy of Oujda, Mohammed First University of Oujda, Oujda, MAR; 3 Medical Oncology, Mohammed VI University Hospital, Oujda, MAR

**Keywords:** case report, encephalopathy, nasopharyngeal, carcinoma, cisplatin

## Abstract

Cisplatin is a cancer therapy drug commonly used. It is well-known for its antineoplastic properties, as well as for its numerous adverse effects, particularly its neurotoxicity. Symptoms associated with a central nervous system injury are unusual but can present a diagnostic challenge. Here, we report a case of a 62-year-old patient who was diagnosed with undifferentiated nasopharyngeal carcinoma. Cisplatin-based chemotherapy was administrated. Five days following the second cycle of treatment, the patient presented neurological disorders. A full biological workup and brain imaging were requested and revealed no abnormalities. The diagnosis of cisplatin encephalopathy was then suspected. Twenty days after cessation of cisplatin therapy, the neurological symptoms began to improve. Based on our case and a review of the literature, cisplatin-induced encephalopathy remains unusual. Its diagnosis is based on a combination of clinical, biological, and radiological criteria and requires the exclusion of other etiologies for neurological disorders in a patient being treated for cancer. Treatment is symptomatic and depends on stopping cisplatin therapy. These neurological adverse effects are often transitory and disappear without major repercussions.

## Introduction

Cisplatin is an alkylating agent widely used in the treatment of several types of cancers, including leukemia, lymphoma, bladder, testicular, ovarian, head and neck, cervical cancer, and sarcoma [1]. Cisplatin's antineoplastic properties are primarily a result of its capacity to cross-link with DNA, preventing transcription and replication [2]. Cisplatin is frequently used in several settings, including concurrent chemoradiotherapy [3].

The use of cisplatin has many side effects. Nephrotoxicity is the most typical adverse side effect of cisplatin. Ototoxicity, neurotoxicity, gastrointestinal toxicity, and hematological toxicity are also common [2]. In most cases, cisplatin often induces peripheral neuropathy. The central nervous system can occasionally be involved [4,5].

In this review, we present, to the best of our knowledge, the first reported case in Morocco of a patient who received cisplatin-based treatment for undifferentiated nasopharyngeal carcinoma and developed a cisplatin-induced encephalopathy.

## Case presentation

We report the case of a 62-year-old woman, followed for diabetes, hypertension, and dyslipidemia, referred to our hospital for the management of locally advanced undifferentiated nasopharyngeal carcinoma, classified cT2N3M0. Following discussion at the multidisciplinary team meeting, the decision was made to put the patient on induction chemotherapy with cisplatin (80 mg/m^2^, day 1, i.v) and gemcitabine (1000 mg/m^2^, day 1, 8 i.v), (J1=J21), followed by concurrent chemoradiotherapy.

Five days after the second cycle of treatment, the patient complained of a severe headache, memory loss, and confusion but no seizures.

**Figure 1:**
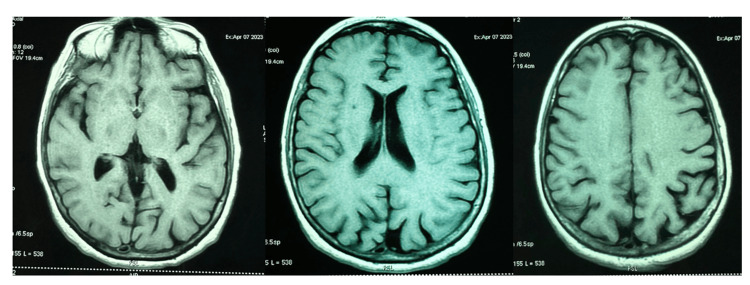
Magnetic resonance imaging (MRI) of the brain, axial T1-weighted image, showing no abnormalities

**Figure 2:**
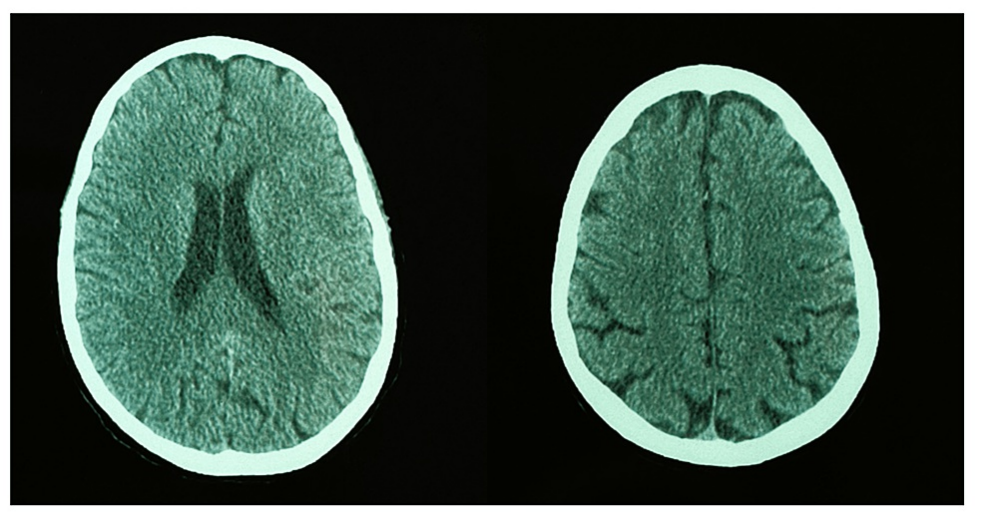
Normal computed tomography (CT) scan in axial cerebral reconstruction

On admission, the patient was confused, had a Glasgow Coma Scale (GCS) of 14/15, was hemodynamically and respiratory stable, apyretic, and had a correct blood sugar level. During the ear, nose, and throat (ENT) examination, a 3-cm right laterocervical adenopathy, well-limited and fixed, was identified. The neurological examination indicated a disoriented patient. There was no neck rigidity or sensory or motor deficit. The cranial nerve examination was unremarkable The rest of the neurological examination was normal. A complete blood count (CBC) was requested and the results revealed no abnormalities, including normal levels of natremia (Na+ =140 mmol/L; normal range: 135-145 mmol/L), corrected calcemia (89 mg/L; normal range: 85-105 mg/L), magnesemia (0.8 mmol/L; normal range: 0.7 and 1.1 mmol/L), kaliemia (3.6 mmol/L; normal range: 3.5 and 5 mmol/L) and fasting blood sugar level (1.19 g/L; normal range: 0.7-1). Tests of kidney and liver function were likewise unremarkable. Both angio brain magnetic resonance imaging (MRI) (Figure 1) and cerebral computed tomography (CT) (Figure 2) were normal.

The diagnosis of cisplatin encephalopathy was then suspected. The patient was hospitalized for five days and was subsequently placed on isotonic saline rehydration and the cisplatin therapy was stopped. Twenty days following the end of cisplatin treatment, the neurological symptoms began to improve, which served as a marker for the evolution.

The case was discussed in our cancer multi-disciplinary team meeting. The patient would receive concomitant docetaxel-based chemoradiotherapy followed by capecitabine-based adjuvant chemotherapy. After one year of follow-up, the patient is still under control and has shown improvement in both clinical and radiological response. Throughout the one-year follow-up period, the patient has not experienced another incident of drug-induced encephalopathy.

## Discussion

Cisplatin is a drug widely used in oncology. It has a large spectrum of adverse effects in addition to its anticancer properties, especially nephrotoxicity, which is a major adverse effect; gastrointestinal disorders, cytotoxicity, ototoxicity, neurotoxicity, and hepatotoxicity [6,7].

The neurotoxicity mostly affects the peripheral nervous system. It can be explained by the higher susceptibility of platinum drugs to access the dorsal root ganglia and peripheral nerves, in comparison to the central nervous system (CNS) [7,8].

CNS toxicity due to cisplatin remains rare. Only a few cases have been reported in the literature. Since it was first documented in 1980, only 17 cases of cisplatin-induced encephalopathy have been observed [9-11]. Today, its global incidence is not known due to its rarity [12].

The first case of cisplatin-induced encephalopathy was reported in 1980 by Berman et al. [13]. The case was of a 30-year-old patient treated for a disseminated testicular embryonal-cell carcinoma with cisplatin, bleomycin, and vinblastine-based chemotherapy. The patient presented nausea and loss of vision on the day of therapy. He then developed seizures. His full biological workup and brain imaging were normal. After symptomatic treatment, which included isotonic saline rehydration, parenteral antiemetic, anticonvulsant treatment, and cisplatin discontinuation, the evolution was marked by an improvement in neurological symptomatology [13].

For diagnosis, there have been reports of two main types of CNS disorders. Posterior reversible encephalopathy syndrome (PRES) is the most common, platinum-associated CNS damage documented in the literature. Clinical symptoms of PRES may include cortical blindness, headache, convulsions, diminished awareness, and hypertension [9,12]. Additionally, these patients have specific radiological abnormalities; punctate or confluent areas of elevated signal on proton density T2-weighted, and fluid-attenuated inversion recovery (FLAIR) images are the most typical MRI abnormalities, with an occipital lobe predominance. However, it is crucial to remember that any part of the CNS might be impacted. Other individuals, like our patient, have localized neurological disorders with or without seizures [5,9,14,15]. The symptoms of this second type of CNS illness appear between a few hours and three months following the last cisplatin exposure and are unrelated to the cumulative dosage of cisplatin. Cerebral CT and MRI are usually normal. Occipital and frontal cortical abnormalities, as well as cerebral atrophy or reversible cerebral edema, are occasionally seen [5,16]. Cerebrospinal fluid (CFS) studies are either normal or indicate only non-specific alterations such as increased protein levels and high opening pressure [5,17]. Even after repeated instances, the symptomatology usually disappears without leaving any permanent neurological damage [9,12,18]. Similarly, our patient presented with an apyretic neurological disorder including memory loss and confusion without seizures, five days after cisplatin exposure with a normal blood test and normal cerebral imaging (including cerebral CT scan and angio-brain MRI). After the exclusion of metabolic causes, infectious causes, and metastatic involvement, the diagnosis of cisplatin-induced encephalopathy was retained.

Thus, cisplatin-induced encephalopathy is diagnosed using a multidisciplinary approach that includes clinical, radiological, and biological criteria. We must take into account the occurrence of neurological symptoms shortly following cisplatin infusion and rule out the other causes of encephalopathy in patients with cancer such as brain metastases, infectious causes, cerebral hemorrhage, and metabolic disorders (hyper/hypoglycemia, hyper/hypocalcemia, hyper/hyponatremia, azotemia) [5,8]. The improvement in symptomatology a few days after discontinuing therapy further supports the diagnosis.

Management of cisplatin-induced encephalopathy is mainly based on discontinuation of cisplatin therapy and supportive measures. Hydration, supportive care, anticonvulsant medication, and correction of hyperthermia and metabolic abnormalities, notably hypomagnesemia, are the main tenets of this approach [5,18,19].

In our case, the patient received symptomatic treatment, which consisted of fluid support, parenteral nutrition, and cessation of cisplatin therapy, since the patient did not present any seizures or electrolyte disorders. Twenty days later, the patient improved her cognitive function without any neurological damage.

Despite the serious clinical symptoms, the neurological symptomatology often regresses in the days following the end of cisplatin treatment and disappears without any repercussions, and patients recover completely from cisplatin-induced encephalopathy in fewer than three weeks of treatment [9,20-22].

The resumption of cisplatin therapy is still a controversial matter. Many argue that cisplatin encephalopathy is not an absolute contraindication to reintroducing platinum salts and that this choice should always be discussed at a cancer multidisciplinary team meeting while considering other clinical aspects [5,10,11].

Concurrent chemoradiotherapy (CCRT) with platinum salts, especially cisplatin, remains a cornerstone in the treatment of ENT malignancies, particularly nasopharyngeal carcinoma [23]. The use of other drugs instead of cisplatin has not been the subject of many investigations.

In our case, three options were available for the therapeutic project: continue the treatment with radiation alone and rechallenge with platinum salts concomitantly with radiotherapy with the risk of a recurrence of platinum-induced encephalopathy or treat the patient with concurrent CCRT while using other drugs.

For the first option, a retrospective study was done to compare radiotherapy alone versus concurrent chemoradiotherapy. This study concluded that radiotherapy alone can therefore be a treatment option, however, its effectiveness is limited [24].

Rechallenge with platinum-based chemotherapy may also be a therapeutic option. In fact, numerous cases of cisplatin encephalopathy documented in the literature have been retreated with platinum drugs [5,25,26].

In terms of the third therapeutic alternative for our patient, phase I trials and retrospective trials have been conducted using other chemotherapeutic drugs concurrently with radiation such as weekly docetaxel and weekly paclitaxel or gemcitabine [27,28]. These trials have shown that these drugs can be an effective and well-tolerated therapeutic option [29].

Our case was discussed in the cancer multidisciplinary team meeting; the patient would receive docetaxel concurrently with radiotherapy and capecitabine-based adjuvant chemotherapy.

Today, thanks to appropriate treatment and successful management, our patient is still under control and has not experienced another incident of drug-induced encephalopathy throughout the control period.

## Conclusions

In summary, cisplatin-induced encephalopathy is still uncommon. However, physicians need to remember that the development of central neurological symptoms in a patient treated for cancer may not always be due to central nervous system invasion; sometimes, they might be drug-related. These neurological side effects are usually transitory and reversible if cisplatin therapy is stopped. Even after repeated episodes of cisplatin-induced encephalopathy, the majority of individuals recover completely without consequences. The decision to discontinue or reintroduce cisplatin therapy after neurological side effects is still a controversial matter and should be made in multidisciplinary team meetings.

## References

[1] Brown A, Kumar S, Tchounwou PB (2019). Cisplatin-based chemotherapy of human cancers. J Cancer Sci Ther.

[2] Qi L, Luo Q, Zhang Y, Jia F, Zhao Y, Wang F (2019). Advances in toxicological research of the anticancer drug cisplatin. Chem Res Toxicol.

[3] Iqbal MS, Chaw C, Kovarik J (2017). Primary concurrent chemoradiation in head and neck cancers with weekly cisplatin chemotherapy: analysis of compliance, toxicity and survival. Int Arch Otorhinolaryngol.

[4] Sioka C, Kyritsis AP (2009). Central and peripheral nervous system toxicity of common chemotherapeutic agents. Cancer Chemother Pharmacol.

[5] Lyass O, Lossos A, Hubert A, Gips M, Peretz T (1998). Cisplatin-induced non-convulsive encephalopathy. Anticancer Drugs.

[6] Rancoule C, Guy JB, Vallard A, Ben Mrad M, Rehailia A, Magné N (2017). 50th anniversary of cisplatin [Article in French]. Bull Cancer.

[7] Astolfi L, Ghiselli S, Guaran V (2013). Correlation of adverse effects of cisplatin administration in patients affected by solid tumours: a retrospective evaluation. Oncol Rep.

[8] Dana R, Spartacus RK, Mutha S, Bhat P (2016). Seizure following chemotherapy (paclitaxel and cisplatin) in a patient of carcinoma cervix. Indian J Pharmacol.

[9] Steeghs N, de Jongh FE, Sillevis Smitt PA, van den Bent MJ (2003). Cisplatin-induced encephalopathy and seizures. Anticancer Drugs.

[10] Mead GM, Arnold AM, Green JA, Macbeth FR, Williams CJ, Whitehouse JM (1982). Epileptic seizures associated with cisplatin administration. Cancer Treat Rep.

[11] Gorman DJ, Kefford R, Stuart-Harris R (1989). Focal encephalopathy after cisplatin therapy. Med J Aust.

[12] Kabre RS, Kamble KM (2016). Gemcitabine and cisplatin induced posterior reversible encephalopathy syndrome: a case report with review of literature. J Res Pharm Pract.

[13] Berman IJ, Mann MP Seizures and transient cortical blindness associated with cis-platinum (II) diamminedichloride (PDD) therapy in a thirty-year-old man. Cancer.

[14] Di Genova L, Perruccio K, Celani MG, Mastrodicasa E, Cantisani TA, Esposito S, Caniglia M (2019). Chemotherapy-related encephalopathy with super-refractory status epilepticus in a child with osteosarcoma: a case report with a review of literature. Front Pharmacol.

[15] Bellin SL, Selim M (1988). Cisplatin-induced hypomagnesemia with seizures: a case report and review of the literature. Gynecol Oncol.

[16] Young DC, Mitchell A, Kessler J, Christman JE (1993). Cortical blindness and seizures possibly related to cisplatin, vinblastine, and bleomycin treatment of ovarian dysgerminoma. J Am Osteopath Assoc.

[17] Cohen RJ, Cuneo RA, Cruciger MP, Jackman AE (1983). Transient left homonymous hemianopsia and encephalopathy following treatment of testicular carcinoma with cisplatinum, vinblastine, and bleomycin. J Clin Oncol.

[18] Vihinen PP, Kätkä KM, Johansson RK, Vihinen TA, Salminen EK (2003). Acute reversible encephalopathy after repeated low-dose cisplatin infusions and concomitant radiotherapy for cancer of the tongue. Acta Oncol.

[19] Sterzing F, Grehn C, Dinkel J, Krempien R, Hartung G, Debus J, Harms W (2007). Severe reversible toxic encephalopathy induced by cisplatin in a patient with cervical carcinoma receiving combined radiochemotherapy. Strahlenther Onkol.

[20] Cherniawsky H, Merchant N, Sawyer M, Ho M (2017). A case report of posterior reversible encephalopathy syndrome in a patient receiving gemcitabine and cisplatin. Medicine (Baltimore).

[21] Zahir MN, Masood N, Shabbir-Moosajee M (2012). Cisplatin-induced posterior reversible encephalopathy syndrome and successful re-treatment in a patient with non-seminomatous germ cell tumor: a case report. J Med Case Rep.

[22] Verschraegen C, Conrad CA, Hong WK (1995). Subacute encephalopathic toxicity of cisplatin. Lung Cancer.

[23] Hennequin C, Guillerm S, Quero L (2019). Combination of chemotherapy and radiotherapy: a thirty years evolution. Cancer Radiother.

[24] Katano A, Takahashi W, Yamashita H (2018). Radiotherapy alone and with concurrent chemotherapy for nasopharyngeal carcinoma. A retrospective study. Medicine (Baltimore).

[25] Wu X, Huang PY, Peng PJ (2013). Long-term follow-up of a phase III study comparing radiotherapy with or without weekly oxaliplatin for locoregionally advanced nasopharyngeal carcinoma. Ann Oncol.

[26] Dechaphunkul T, Pruegsanusak K, Sangthawan D, Sunpaweravong P (2011). Concurrent chemoradiotherapy with carboplatin followed by carboplatin and 5-fluorouracil in locally advanced nasopharyngeal carcinoma. Head Neck Oncol.

[27] Hoffmann W, Belka C, Schmidberger H, Budach W, Bochtler H, Hess CF, Bamberg M (1997). Radiotherapy and concomitant weekly 1-hour infusion of paclitaxel in the treatment of head and neck cancer—results from a phase I trial. Int J Radiat Oncol Biol Phys.

[28] Eisbruch A, Shewach DS, Bradford CR (2001). Radiation concurrent with gemcitabine for locally advanced head and neck cancer: a phase I trial and intracellular drug incorporation study. J Clin Oncol.

[29] Nakahara S, Hanamoto A, Seo Y (2016). Chemoradiotherapy with weekly low-dose docetaxel and cisplatin concurrent with radiation for patients with locally advanced nasopharyngeal carcinoma, followed by adjuvant chemotherapy for selected patients. Jpn J Clin Oncol.

